# Validation of a one degree-of-freedom spherical model for kinematics analysis of the human ankle joint

**DOI:** 10.1186/1757-1146-5-S1-P13

**Published:** 2012-04-10

**Authors:** Nicola Sancisi, Vincenzo Parenti-Castelli, Benedetta Baldisserri, Claudio Belvedere, Matteo Romagnoli, Valentina D’Angeli, Alberto Leardini

**Affiliations:** 1 Department of Mechanical Engineering-DIEM, University of Bologna, 40136 Bologna, Italy; 2 Movement Analysis Laboratory, Istituto Ortopedico Rizzoli, 40136 Bologna, Italy

## Background

During passive motion, the human tibiotalar (ankle) joint behaves as a single degree-of-freedom (1DOF) system [1,2]. In these conditions, fibres within the ligaments remain nearly isometric throughout the flexion arc and articular surfaces nearly rigid. Relevant theoretical models are showing that the ligaments and the articular surfaces act together as mechanisms to control the passive joint kinematics [3-5]. Kinematic measurements and corresponding model predictions also revealed that the instantaneous screw axes of passive motion pass near to a single point, hereinafter called pivot point [5]. The present study investigates the extent to which this motion is spherical-like.

## Materials and methods

A 1DOF Spherical Parallel Mechanism is analyzed, based both on joint anatomy and kinematics: the calcaneal-fibular and tibio-calcaneal ligaments are modelled as binary links of constant length, and relevant bones are connected by a spherical pair centred at the pivot point [5]. Geometrical data and reference motion were obtained from experiments in 5 amputated lower limbs, free from anatomical defects. Anatomical landmarks, articular surfaces and ligament origins and insertions were digitized. Passive dorsi-/plantar-flexion cycles were performed and relevant bone motion was recorded by a standard stereo-photogrammetric device. The pivot point was obtained by searching the point with the least mean squared distance from the instantaneous screw axes of passive motion. The closure equations were solved to obtain the simulated motion of the joints, to compare it with the original experimental motion.

## Results

**Figure 1:**
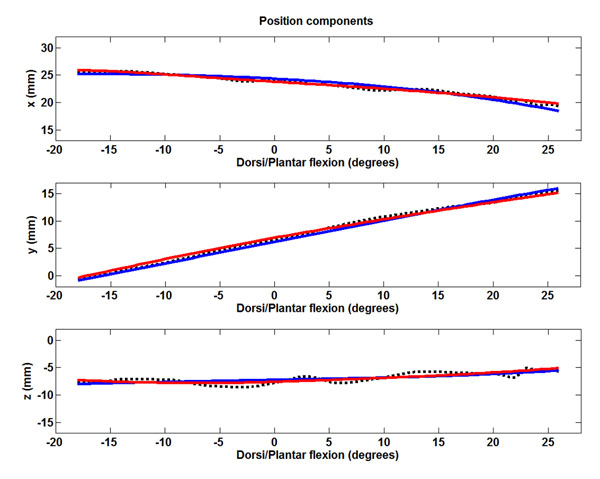
The three displacements of a typical specimen, obtained from experiments (black), a previous model (red) and the spherical one (blue).

In all specimens, the model replicated passive motion with a very good precision (Figure 1).

## Conclusions

The passive motion of the ankle joints can be approximated well by a 1DOF spherical mechanism, despite the simple structure of this model. Replication of the original experimental motion can be a little worse than using previous mechanisms [4] (Figure 1), but computational costs, mechanical complexity and numerical instabilities are significantly reduced.
